# Time-series analysis of temperature variability and cardiovascular emergency department visits in Atlanta over a 27-year period

**DOI:** 10.1186/s12940-024-01048-4

**Published:** 2024-01-23

**Authors:** Morgan Lane, Stefanie Ebelt, Zhen Wu, Noah Scovronick, Rohan R. D’Souza, Howard H. Chang

**Affiliations:** 1 Gangarosa Department of Environmental Health, Emory University, 1518 Clifton Rd, Atlanta, GA USA; 2 Department of Biostatistics and Bioinformatics, Emory University, 1518 Clifton Rd, Atlanta, GA USA

**Keywords:** Temperature variability (TV), Cardiovascular morbidity, Emergency department, Adult population

## Abstract

**Background:**

Short-term temperature variability, defined as the temperature range occurring within a short time span at a given location, appears to be increasing with climate change. Such variation in temperature may influence acute health outcomes, especially cardiovascular diseases (CVD). Most research on temperature variability has focused on the impact of within-day diurnal temperature range, but temperature variability over a period of a few days may also be health-relevant through its impact on thermoregulation and autonomic cardiac functioning. To address this research gap, this study utilized a database of emergency department (ED) visits for a variety of cardiovascular health outcomes over a 27-year period to investigate the influence of three-day temperature variability on CVD.

**Methods:**

For the period of 1993–2019, we analyzed over 12 million CVD ED visits in Atlanta using a Poisson log-linear model with overdispersion. Temperature variability was defined as the standard deviation of the minimum and maximum temperatures during the current day and the previous two days. We controlled for mean temperature, dew point temperature, long-term time trends, federal holidays, and day of week. We stratified the analysis by age group, season, and decade.

**Results:**

All cardiovascular outcomes assessed, except for hypertension, were positively associated with increasing temperature variability, with the strongest effects observed for stroke and peripheral vascular disease. In stratified analyses, adverse associations with temperature variability were consistently highest in the moderate-temperature season (October and March-May) and in the 65 + age group for all outcomes.

**Conclusions:**

Our results suggest that CVD morbidity is impacted by short-term temperature variability, and that patients aged 65 and older are at increased risk. These effects were more pronounced in the moderate-temperature season and are likely driven by the Spring season in Atlanta. Public health practitioners and patient care providers can use this knowledge to better prepare patients during seasons with high temperature variability or ahead of large shifts in temperature.

**Supplementary Information:**

The online version contains supplementary material available at 10.1186/s12940-024-01048-4.

## Background

As the climate changes and weather patterns are altered [1], it is important to understand how these fluctuations can impact human health. One characteristic of altered weather patterns is increased temperature variability (TV), which can be defined on a narrow timescale, such as within 24 h, or on longer timescales, such as over a few days, or even longer periods of weeks, months, or years. TV over shorter periods of time (inter- or intra-day) can have acute impacts on health [2, 3]; existing epidemiologic evidence indicates that cardiovascular disease (CVD) health outcomes are associated with short-term changes in temperature and TV [4–8], with effects varying across age groups, gender, and other individual and area-level factors [9, 10]. These outcomes may be associated with the impact of temperature extremes on thermoregulation, increasing activity in the autonomic nervous system and leading to changes in cardiac functioning [2, 9].

Research on TV and health has predominantly focused on diurnal temperature range (DTR), with high diurnal variability shown to increase cardiovascular mortality and morbidity [6, 11–15]. There is evidence, however, that multi-day TV may also have important associations with cardiovascular health [16–20]. Most of the research in this area has focused on mortality, and many morbidity studies that investigate the influence of weather patterns are restricted to the elderly population with a focus on the severe outcome of hospitalization, with a large focus on specific regions such as Europe, China, and the Northeastern United States [10, 12–15, 18]. To strengthen public health preparedness, it is important to investigate cardiovascular morbidity across age groups and severity levels, as well as in unique regions.

This study uses emergency department (ED) visit data for a variety of CVD health outcomes from hospitals in Atlanta during 1993–2019. This uniquely extensive dataset allows us to investigate the effects of TV on an understudied measure of cardiovascular morbidity in a temperate city, to examine whether effects vary across seasons and age groups, and is the first study to our knowledge to assess whether effects vary by decade.

## Methods

### Health data

Patient-level daily ED visit data for seven cardiovascular outcomes were collected from individual hospitals for the years 1993 to 2013, and from the Georgia Hospital Association for the years 2014 to 2019, for facilities located in the 20-county metro Atlanta area [21]. The definition of an ED visit included patients who visited the ED and were then discharged directly, as well as ED patients admitted to the hospital. International Classification of Diseases (ICD) 9th revision (ICD-9) diagnosis codes were used for ED visits prior to October 1, 2015, and ICD 10th revision (ICD-10) codes were used for the rest of the period. We identified cause-specific ED visits using both the primary and secondary diagnosis codes in Table 1. Daily visit counts for each CVD outcome were aggregated by date and residential ZIP code, as well as by age group (0–19, 19–64 and 65 + years).

**Table 1:** ICD-9 and ICD-10 codes used to identify cause-specific ED visits

Outcome	ICD-9 Codes	ICD-10 Codes
Hypertension	401–405	I10-I15
Ischemic Heart Disease	410–414	I20-I25
Dysrhythmia	427	I46-I49
Congestive Heart Failure	428	I50
Stroke	433–437	G45, I63-I67
Peripheral Vascular Disease	440–447	I70-I79
Myocardial Infarction	410	I21-I22
Combined Outcomes	All ICD-9 codes listed above	All ICD-10 codes listed above

### Meteorologic data

Daily meteorological data were obtained from the National Centers for Environmental Information from the automated surface observing station located at the Atlanta Hartsfield International Airport. The data included daily minimum, maximum, and mean temperature, and dew point temperature.

TV was defined as the standard deviation (SD) of the minimum and maximum temperatures during the current day and the previous two days: TV_0–2_ = SD (MinTemp_lag0_, MaxTemp_lag0_, MinTemp_lag−1_, MaxTemp_lag−1_, MinTemp_lag−2_, MaxTemp_lag−2_). We restricted TV to a 3-day exposure period because of the a priori focus of this analysis on short-term temperature variability. The inclusion of the minimum and maximum temperature across multiple days allowed us to account for both intra- and inter-day variability.

### Statistical analysis

The association between TV and cardiovascular ED visits was estimated using a Poisson log-linear model with overdispersion. A linear relationship between TV and morbidity was assumed based on previous work in mortality [16]. Long-term trends in demographics and other community characteristics, as well as seasonality, were controlled for using a natural cubic spline with 12 degrees of freedom. Dew point temperature and the three-day moving average of mean temperature were controlled for using splines with 6 degrees of freedom. We also controlled for Federal holidays and day of the week using indicator variables. We report the relative risk (RR) of an ED visit associated with an interquartile range (IQR) increase in TV.

To examine whether the effects of TV differed by season, age group, or decade, we conducted stratified analyses. We conducted a seasonality analysis to compare associations across three seasons: warm (June to September), moderate (March to May and October), and cold (November to February). We chose to conduct a three-season analysis because we hypothesized that these ‘shoulder’ seasons might differentially impact individuals in Atlanta given these are the times when air conditioning use may be less consistent as the seasons shift and temperature variability is changing (decreasing from the winter and increasing from the summer). We defined each season period using average monthly temperature over the analysis time period. We also conducted a four-season analysis: winter (December to February), spring (March to May), fall (September to November), and summer (June to August). Additionally, we conducted an age group analysis, comparing those aged 65 and older to those between the ages of 19 and 64. We originally included individuals under the age 19 in the analysis as well, but this group comprised less than 1% of the data rendering comparisons difficult. The decade analysis was split up as follows: 1993–1999, 2000–2009, and 2010–2019. Additionally, we conducted a decade analysis stratified by season.

Sensitivity analyses were conducted to assess whether associations between TV and ED visits were robust when varying the degrees of freedom for the long-term trend (from 6 to 12 *df*), the effect of three-day moving average of mean temperature (6–8 *df*), and the effect of dew point temperature (6–8 *df*) controls. In addition, we examined the use of 3-day moving average of minimum or maximum temperature to control for effects of absolute temperature instead of mean temperature. In another sensitivity analysis, we restricted the analyses to only cardiovascular ED visits identified by the primary diagnosis. We also conducted one other seasonality analyses for two seasons: warm (May to September) and cold (January to April and October to December). Finally, we examined other definitions for TV that use [1] a different set of temperature variables (minimum, maximum, and mean), and [2] a different length of exposure period (two days) to calculate the standard deviation.

Analyses were performed in R Software (Version 4.1.2) with the package “splines” for creating natural cubic splines.

## Results

Our study included 12,281,210 cardiovascular ED visits during 1993–2019. Table 2 shows the total number of ED visits for each cardiovascular outcome by age group. ED visits for hypertension constituted the largest portion of visits across the study period. Patients 65 and over made up the largest portion of visits for all health outcomes except hypertension. Table S1 shows ED visit counts by season, by diagnosis type (primary versus secondary), and by decade. The majority of cases (85%) were ascertained by secondary diagnosis codes, except for stroke and myocardial infarction, and the number of cases did not vary greatly between seasons. Table 3 shows the summary statistics for minimum, maximum, and mean temperature, and TV across the study period by season and overall. TV was similar in the moderate and cold seasons and lowest in the warm season. There was also in increase in TV in more recent years.

**Table 2:** Total and mean number of emergency department visits in the Atlanta metropolitan area from 1993–2019 for each health outcome by age group ascertained using both primary and secondary diagnosis code

Health Outcome	Age Group	Number of Visits (%)	Mean (SD)
Combined Outcomes	Ages 0–18	47,515 (0.4)	2 (2)
	Ages 19–64	6,485,482 (53)	658 (490)
	Ages 65+	5,748,213 (47)	291 (223)
Hypertension	Ages 0–18	21,830 (0.3)	1 (1)
	Ages 19–64	4,516,397 (62)	115 (264)
	Ages 65+	2,809,013 (38)	142 (113)
Ischemic Heart Disease	Ages 0–18	797 (0)	0.03 (0.1)
	Ages 19–64	711,618 (42)	72 (50)
	Ages 65+	985,600 (58)	50 (37)
Dysrhythmia	Ages 0–18	17,889 (1)	0.6 (1)
	Ages 19–64	461,407 (36)	48 (31)
	Ages 65+	817,660 (63)	41 (34)
Congestive Heart Failure	Ages 0–18	2,990 (0.2)	0.1 (0.3)
	Ages 19–64	514,091 (43)	52 (46)
	Ages 65+	693,897 (57)	35 (29)
Stroke	Ages 0–18	1,893 (0.6)	0.06 (0.3)
	Ages 19–64	111,175 (37)	11 (8)
	Ages 65+	186,492(62)	9 (7)
Peripheral Heart Disease	Ages 0–18	1,969 (0.8)	0.07 (0.3)
	Ages 19–64	89,676 (35)	9 (7)
	Ages 65+	162,571 (64)	8 (7)
Myocardial Infarction	Ages 0–18	147 (0.1)	0 (0.07)
	Ages 19–64	81,118 (47)	8 (5)
	Ages 65+	92,980 (53)	5 (3)

**Table 3:** Daily mean, standard deviation (SD), and interquartile range (IQR) of minimum, maximum, and mean temperature, and temperature variability in Atlanta from 1993–2019 by season and over the full period. The cold season is defined as November to February, the moderate season is defined as March to May and October, and the warm season is defined as June to September

Temperature Variable	Decade	Overall			Cold			Moderate			Warm		
		Mean	SD	IQR	Mean	SD	IQR	Mean	SD	IQR	Mean	SD	IQR
Minimum Temperature (°F)	1993–1999	52.8	15.2	26.2	38.1	10.3	14.1	51.4	10.3	14.4	68.5	5.1	6
	2000–2009	51.9	15.4	27.2	36.5	9.7	13.6	51.1	10	14.9	67.9	4.8	5.1
	2010–2019	53.5	15.5	27	38	10.3	14.1	53	10.1	15	69.5	4.4	4
	**Overall**	**52.7**	**15.4**	**27**	**37.5**	**10.1**	**13.2**	**51.9**	**10.2**	**15.1**	**68.6**	**4.8**	**5.1**
Maximum Temperature (°F)	1993–1999	73.9	14.6	23	59.3	10.1	13.8	74.4	9.7	11.9	87.8	6.3	7.9
	2000–2009	74	13.8	20.9	59.8	9.8	13.8	74.5	8.5	11.9	87.4	5.3	7
	2010–2019	75.8	14.4	22	60.9	10.1	16	76.6	9.2	10.9	89.6	4.9	5.9
	**Overall**	**74.6**	**14.3**	**23.1**	**60.1**	**10**	**13.8**	**75.3**	**9.12**	**12**	**88**	**5.5**	**7**
Mean Temperature (°F)	1993–1999	62.7	14.7	24.5	47.9	9.8	13.3	62.5	9.7	13	77.4	5.3	6.2
	2000–2009	62.3	14.5	24	47.3	9.7	14.5	62.5	9	12.4	76.8	4.7	5.4
	2010–2019	63.8	14.8	24.5	48.5	10.1	14.1	64.3	2.6	13.1	78.5	4.3	5.5
	**Overall**	**63**	**14.7**	**24.4**	**47.9**	**9.9**	**14.1**	**63.1**	**9.4**	**13**	**77.6**	**4.9**	**5.8**
Temperature Variability*	1993–1999	12.3	2.9	3.9	12.7	3.2	4.5	13.3	2.8	3.9	10.9	2	2.7
	2000–2009	12.7	2.8	4.1	13.7	2.8	3.7	13.4	2.7	3.7	10.9	1.7	2.8
	2010–2019	12.8	2.7	3.7	13.5	2.9	3.8	13.6	2.7	3.7	11.2	1.7	2.2
	**Overall**	**12.6**	**2.8**	**3.9**	**13.4**	**3**	**3.9**	**13.4**	**2.7**	**3.7**	**11**	**1.9**	**2.5**

* Temperature variability was defined as the standard deviation of the minimum and maximum temperatures throughout the current day and the previous two days: TV_0–2_ = SD (MinTemp_lag0_, MaxTemp_lag0_, MinTemp_lag-1_, MaxTemp_lag-1_, MinTemp_lag-2_, MaxTemp_lag-2_)

**Fig. 1:**
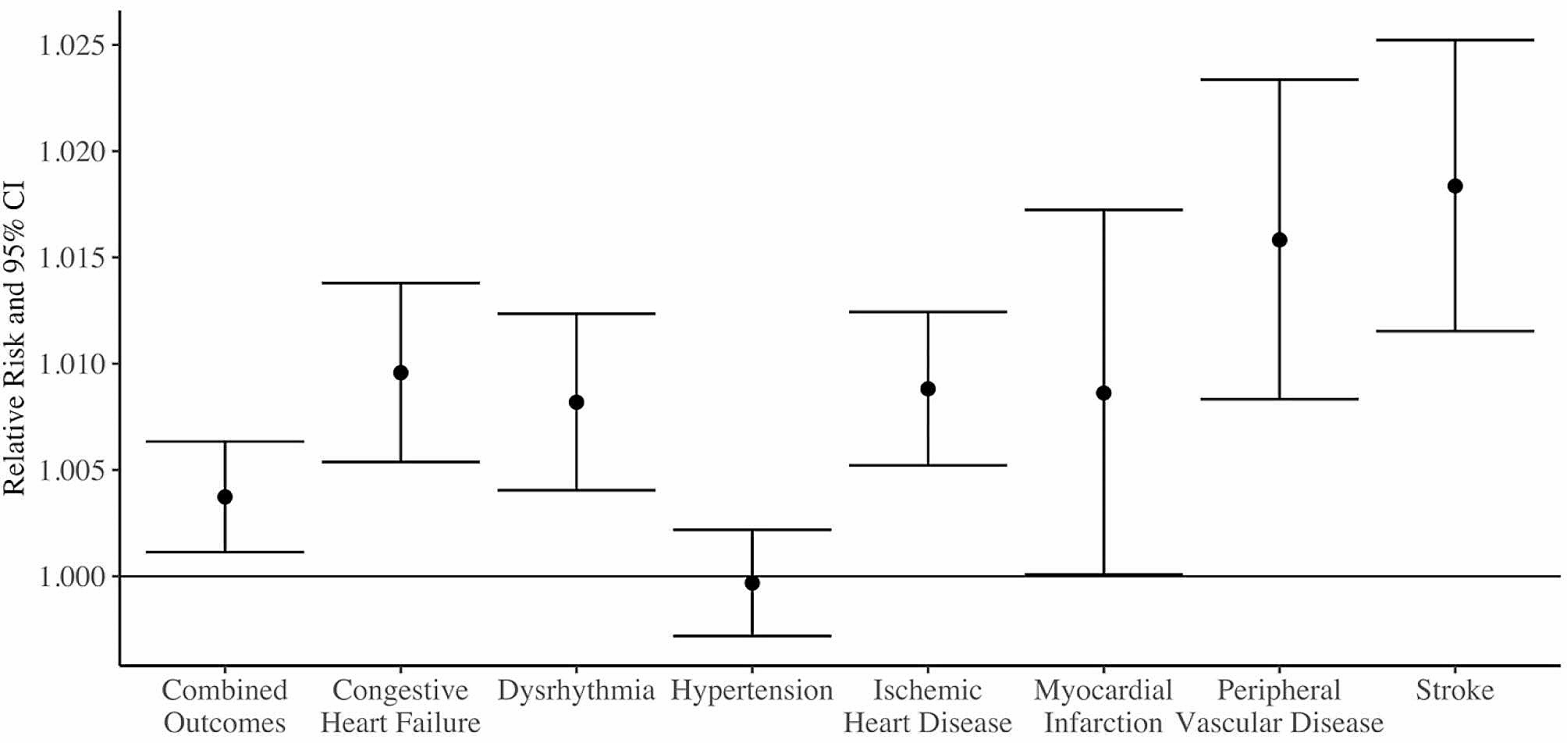
Relative risks and 95% confidence interval of an emergency department visit for each cardiovascular health outcome associated with an interquartile range increase in temperature variability from 1993–2019 in Atlanta, controlling for mean temperature, dew point temperature, time trend, day of the week, and holidays

Figure 1 shows the relative risks of ED visits for each IQR increase in TV for the overall study population for each outcome across the full study period. All cause-specific cardiovascular outcomes except for hypertension show a significant increased relative risk with increasing TV. Stroke (RR: 1.02, 95% CI: 1.01, 1.03) and peripheral vascular disease (RR: 1.02, 95% CI: 1.01, 1.02) showed the highest relative risks, followed closely by the other outcomes.

**Fig. 2:**
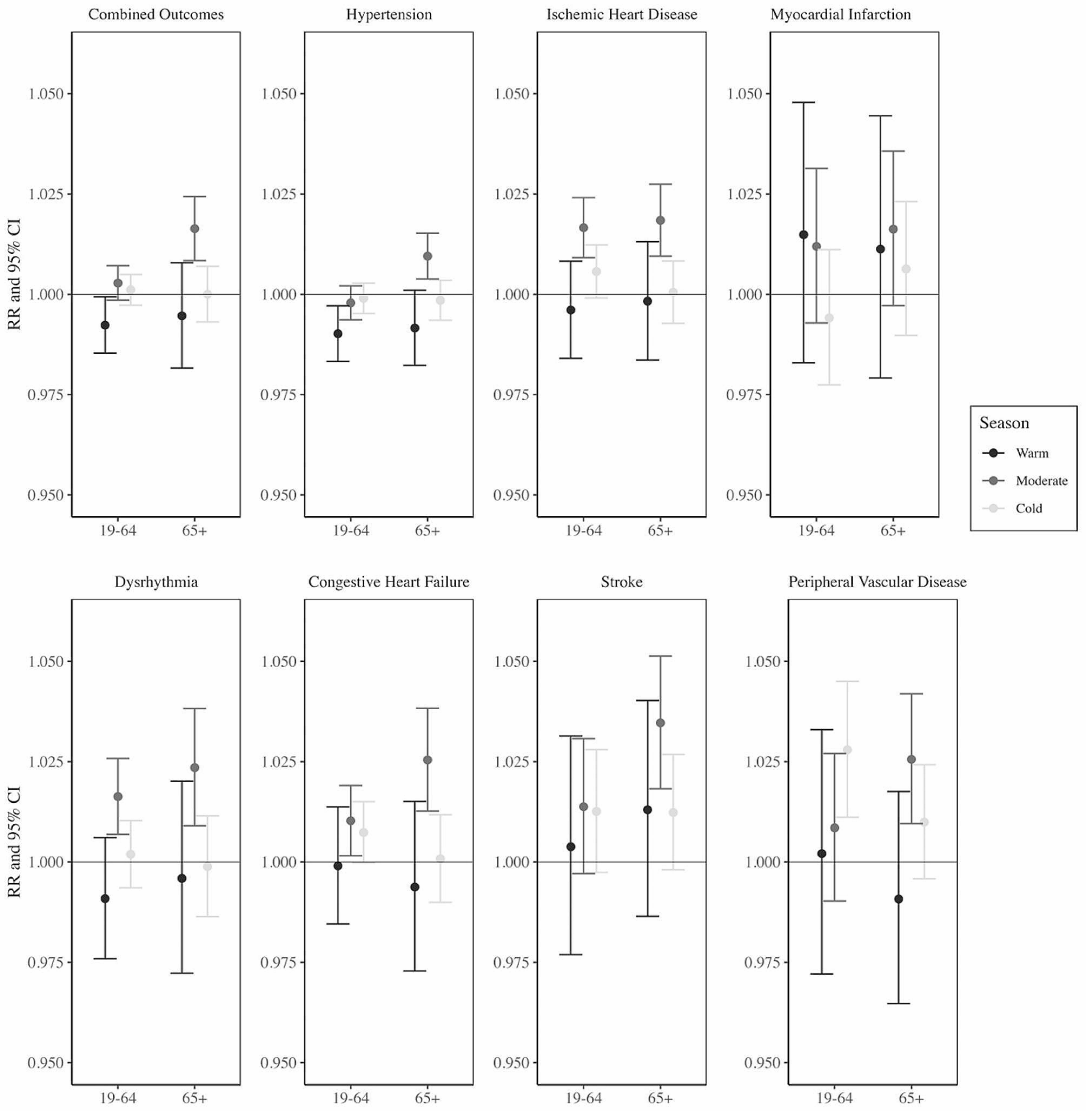
Relative risks and 95% confidence interval of an emergency department visits for each cardiovascular health outcome associated with an interquartile range increase in temperature variability from 1993–2019 in Atlanta, stratified by season (cold, moderate, and warm) and age group (19–64 and 65+), and controlling for mean temperature, dew point temperature, time trend, day of the week, and holidays

Figure 2 presents effect modification by age and season on the TV associations for each outcome. In these stratified analyses, adverse associations with TV were consistently highest in the moderate season and in the 65 + age group for all outcomes. In contrast to the overall analysis (Fig. 1), we found positive associations between ED visits for the combined CVD outcome (RR: 1.02, 95% CI: 1.01, 1.02) and ED visits for hypertension (RR: 1.01, 95% CI: 1.00, 1.02) in the 65 + age group during the moderate season. Associations with stroke ED visits (RR: 1.03, 95% CI: 1.02, 1.05) and peripheral vascular disease ED visits (RR: 1.03, 95% CI: 1.01, 1.04) remained the strongest among cause-specific ED visits in the 65 + age group during the moderate season. The relative risk estimates in the warm and cold seasons were generally similar and null. Estimated associations among the 19–64 age group had large uncertainties possibly due the smaller sample size relative to the 65 + age group for most outcomes.

**Fig. 3:**
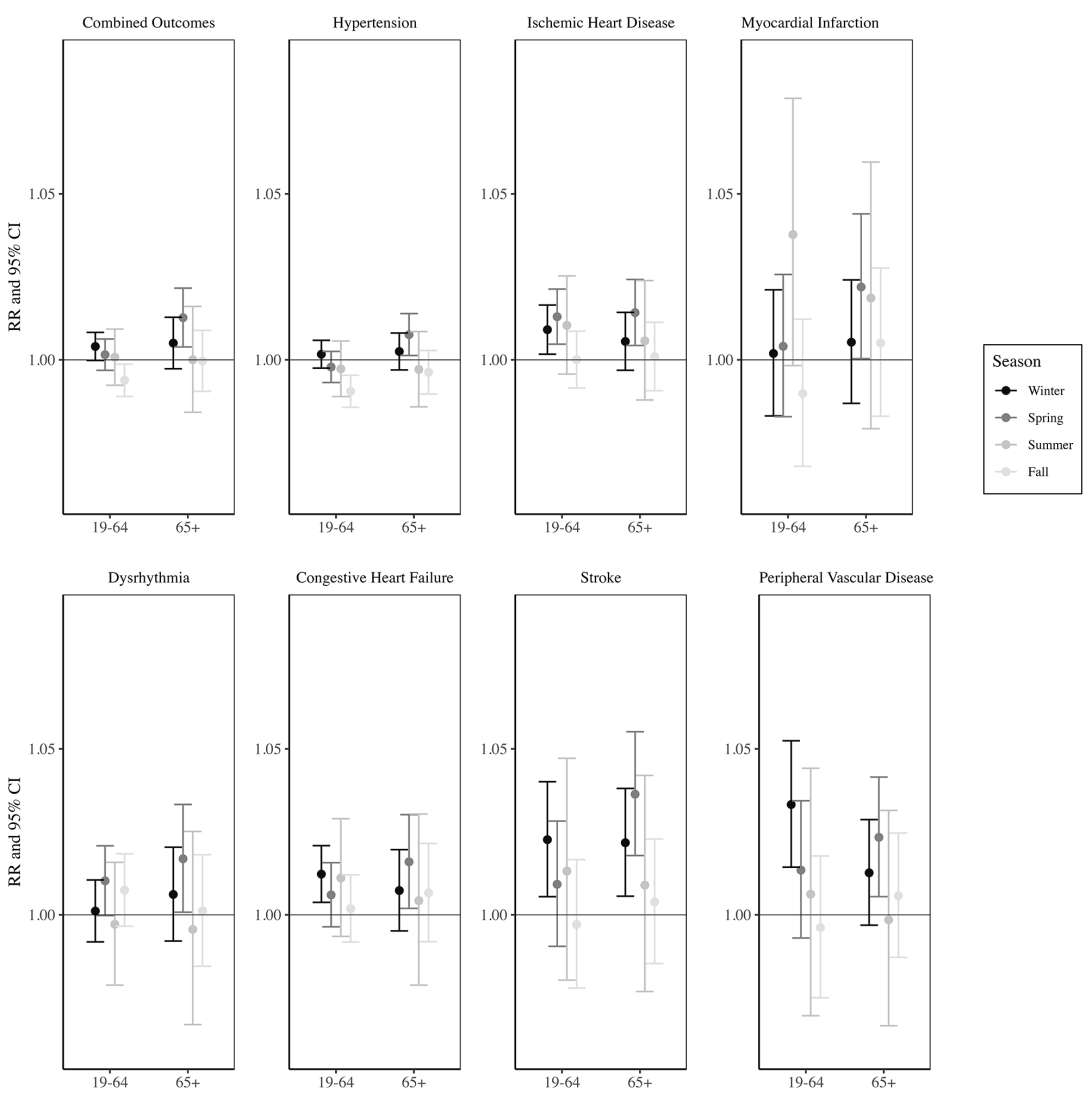
Relative risks and 95% confidence interval of an emergency department visits for each cardiovascular health outcome associated with an interquartile range increase in temperature variability from 1993–2019 in Atlanta, stratified by season (fall, winter, spring, summer) and age group (19–64 and 65+), and controlling for mean temperature, dew point temperature, time trend, day of the week, and holidays

When four seasons were considered, associations were generally strongest for the Spring season (Fig. 3). These results suggest that the Spring season may be the driver of the increased risk estimates for the moderate season in the 3-season analysis (Fig. 2).

When season was defined by two seasons (warm and cold), relative risk estimates were generally similar and null, except for stroke and peripheral vascular disease where the risks were higher and significant in the cold season for those over 65 years of age (Figure S1). When restricting only to cases ascertained from primary diagnoses, the risk estimates for several outcomes (ischemic heart disease, congestive heart failure, stroke, peripheral vascular disease) in the 65 + age group were attenuated, and became consistent with the null except for stroke, compared to the main analysis including primary and secondary diagnoses (Figure S2). When stratifying the analysis by decades, relative risks were highest in the decade 2000–2009 (Figure S3).

In sensitivity analyses, models run with varying degrees of freedom for the time spline showed that 12 degrees of freedom resulted in the lowest AIC values (Figure S4), while changing the degrees of freedom for the exposure spline and the exposure length (two versus three days) resulted no improvement in AIC (Figures S5 and S6). Among the different controls for continuous temperature (mean, minimum, and maximum temperature), the lowest AIC values were for models adjusting for mean temperature (Figure S7). Overall, these sensitivity analyses on different confounder adjustments did not impact estimated associations between ED visits and temperature variability compared to the main model.

## Discussion

All cardiovascular outcomes in this study, except for hypertension, showed increased relative risks of emergency department visits with increasing temperature variability. These effects were stronger in the moderate season, as reported in previous mortality research [16], and may have been driven mostly by the Spring season (Fig. 3) when TV is highest in Atlanta (Table S2). TV in the Spring is more similar to variability in the Winter than in the Fall, but mean temperatures in the Fall are more similar to those in the Spring. We stratified our seasons by mean temperature; therefore, October is included in the moderate season for this analysis. We also decided to stratify this way because October can be considered part of the “shoulder” season in Atlanta, as temperatures begin to drop from summer highs, and air conditioning use might be less consistent, which may have an influence on cardiovascular health. Perhaps the differences in relative risks between the Fall and Spring can be partially explained by differences in behavior for individuals in Atlanta between the two seasons in terms of participation in outdoor activities or use of air conditioning or heating. For some outcomes, the effects were stronger in the cold season than in the warm season, which may be due to lower TV in the warm season. When stratified by age, the associations were stronger in people over 65, as seen in previous research [15].

The lack of an overall association between TV and hypertension ED visits was surprising. Previous research has found that shorter periods of TV are associated with increased hypertension morbidity [22–25]. One possible explanation is that the physiological effects of TV on hypertension are more immediate than the other outcomes examined in this analysis, making DTR a better exposure of interest for this outcome. When running the analysis using DTR as the exposure, the association with hypertension was consistent with the null. While we did not find an overall association with TV and hypertension, when stratified by age and season, we did find an increased risk of hypertension ED visits with increasing TV for individuals over 65 years old in the moderate season when the case definition included visits with either primary or secondary hypertension diagnosis codes, suggesting that TV is a risk factor for hypertension in certain contexts.

Past research has shown that DTR can influence cardiovascular health, and similar physiological explanations may apply here [13, 15]. Specifically, TV may increase cardiovascular work load by impairing thermoregulation, leading to increased heart rate, oxygen uptake, and blood pressure, and may impact the autonomic and sympathetic nervous systems causing impaired cardiac functioning, inflammation, and dehydration [6, 9]. Sympathetic reactivity to temperature changes can affect both acute and chronic conditions and are influenced by comorbid conditions such as cardiac diseases, kidney diseases, and diabetes [26]. There could also be secondary impacts of TV on the environment, such as increased allergens, pathogens, or air pollution, that result in negative cardiovascular outcomes [6]. For this study period, TV was not correlated with daily PM_2.5_ and ozone (correlation coefficient of 0.05, and 0.07, respectively), indicating that air pollution is likely not a confounder or mediator. It is possible as well that swings in temperature may impact individual behaviors such as spending time outdoors or doing physical exercise that may influence cardiovascular health.

In the three-season analysis, we found that the effects of TV on ED visits were strongest in the moderate season, with weaker or null effects in the warm and cold seasons. The observed differences in health effects may have to do with what meteorological factors are driving TV at different times of the year, and how those underlying factors influence health. For example, in the cold season, TV is driven mostly by changes in the maximum temperature, whereas in the warm season, variability is often driven by changes in the minimum temperature [11]. In the spring in Atlanta, high TV exposures are often due to low minimum temperatures in the morning and high temperatures in the afternoon, which is also associated with lower humidity.

Recent studies have found that TV effects on cardiovascular health differ by geographic region, though the evidence is mixed on whether colder or warmer regions have stronger effects [13, 16, 27]. This study found that even in a temperate city with a high prevalence of air conditioning and heating [28], TV was associated with cardiovascular health. The use of air conditioning and heating may also influence the seasonality of the effects of TV if these systems are less commonly utilized in the moderate season, and the indoor climate is less controlled.

One strength of this study is the 27-year data set, the longest time period for an analysis on TV in the United States. This study also contributes to research on TV by using a lesser utilized metric for variability (i.e., considering the standard deviation of minimum and maximum temperatures over a 3-day period, rather than within-day diurnal variation), which provides further insight into the effects of temperature changes across a longer time period than DTR. This analysis also has limitations. We used a single temperature monitoring site to represent TV for the entire metro-Atlanta area. Our use of the single site is supported by the results of our previous time-series study that showed that using population-average exposures derived from one-kilometer temperature data resulted in relative risk estimates similar to those using airport measurement data [29]. The analysis did not account for individual exposures to temperature changes through factors such as access to air conditioning or outdoor occupational exposure, though as mentioned previously, air conditioning is common in Atlanta. Future studies could focus on higher-risk populations, such as individuals with outdoor occupational exposure or those with limited access to indoor climate control. Other individual factors may also influence vulnerability to negative health outcomes from TV such as pre-existing comorbidities or medication use, which were not accounted for in this analysis. Additionally, future research could investigate different types of temperature variability, such as increased variability resulting from a cold or warm front, which could influence individuals’ behaviors differently than increased variability following a long warm season with stable temperatures. There could also be differences in other environmental factors, such as pollen or air pollution, that create joint effects.

## Conclusions

This study highlights the influence that TV over a three-day period can have on cardiovascular morbidity. We show that increasing TV is associated with an increased risk of an ED visit for many cardiovascular outcomes, and that this risk seems to be elevated for people over the age of 65 in the moderate season. Public health practitioners and patient care providers can use this knowledge to better prepare patients during seasons with high TV or ahead of large shifts in temperature. It is important that similar analyses be conducted in other regions that may have different levels of TV, so that the public health response can be tailored for the local climate.

### Electronic supplementary material

Below is the link to the electronic supplementary material.

Supplementary Material 1

## Data Availability

The data that support the findings of this study are available from the Georgia Hospital Association and from individual facilities, but restrictions apply to the availability of these data, which were used under license for the current study, and so are not publicly available. The meteorological data used in this analysis is publicly available from the National Centers for Environmental Information.

## Abbreviations

ED: Emergency Department
TV: Temperature Variability
ICD: International Classification of Diseases
SD: Standard Deviation
CVD: Cardiovascular Outcomes
IQR: Interquartile Range
DTR: Diurnal Temperature range
RR: Relative Risk
