# Supplementary material for: Time-series analysis of temperature variability and cardiovascular emergency department visits in Atlanta over a 27-year period
Source: Environ Health. 2024 Jan 23;23:9. doi: 10.1186/s12940-024-01048-4 (PMC10804549; doi:10.1186/s12940-024-01048-4)
Supplement: Supplementary file 1 — Supplementary Material 1 [file 12940_2024_1048_MOESM1_ESM.docx]

**SUPPLEMENTAL MATERIAL**

**Table S1.** ED Visit counts by season, decade, and type of diagnosis (primary only or primary and secondary combined) for each health outcome.

| **Health Outcome** | **Season** | **Visit Count** | **Percent of Visits** |
| --- | --- | --- | --- |
| Combined Outcomes | Cold | 2,909,199.00 | 33% |
|  | Moderate | 2,949,250.00 | 33% |
|  | Warm | 2,945,833.00 | 33% |
| Hypertension | Cold | 2,423,743.00 | 33% |
|  | Moderate | 2,461,512.00 | 33% |
|  | Warm | 2,463,093.00 | 34% |
| Ischemic Heart Disease | Cold | 564,857.00 | 33% |
|  | Moderate | 571,073.00 | 34% |
|  | Warm | 562,421.00 | 33% |
| Dysrhythmia | Cold | 434,603.00 | 34% |
|  | Moderate | 435,602.00 | 34% |
|  | Warm | 427,113.00 | 33% |
| Congestive Heart Failure | Cold | 413,131.00 | 34% |
|  | Moderate | 407,270.00 | 34% |
|  | Warm | 390,872.00 | 32% |
| Stroke | Cold | 98,733.00 | 33% |
|  | Moderate | 100,882.00 | 34% |
|  | Warm | 100,011.00 | 33% |
| Peripheral Heart Disease | Cold | 84,425.00 | 33% |
|  | Moderate | 86,067.00 | 34% |
|  | Warm | 83,786.00 | 33% |
| Myocardial Infarction | Cold | 60,064.00 | 34% |
|  | Moderate | 58,858.00 | 34% |
|  | Warm | 55,353.00 | 32% |
| **Health Outcome** | **Primary Diagnosis** | **Secondary Diagnosis** | **All Diagnoses** |
| Combined Outcomes | 1,559,543 | 10,721,667.00 | 12,281,210 |
| Hypertension | 517,244 | 6,829,996.00 | 7,347,240 |
| Ischemic Heart Disease | 245,425 | 1,452,590.00 | 1,698,015 |
| Dysrhythmia | 248,136 | 1,048,820.00 | 1,296,956 |
| Congestive Heart Failure | 204,076 | 1,006,902.00 | 1,210,978 |
| Stroke | 200,902 | 98,658.00 | 299,560 |
| Peripheral Heart Disease | 19,472 | 234,744.00 | 254,216 |
| Myocardial Infarction | 124,288 | 49,957.00 | 174,245 |
| **Health Outcome** | **Decade** | **Visit Count** | **Percent of Visits** |
| Combined Outcomes | 1993-1999 | 403,122.00 | 3% |
|  | 2000-2009 | 3,656,667.00 | 30% |
|  | 2010-2019 | 8,223,680.00 | 67% |
| Hypertension | 1993-1999 | 181,402.00 | 2% |
|  | 2000-2009 | 2,138,469.00 | 29% |
|  | 2010-2019 | 5,028,477.00 | 68% |
| Ischemic Heart Disease | 1993-1999 | 75,276.00 | 4% |
|  | 2000-2009 | 532,134.00 | 31% |
|  | 2010-2019 | 1,090,941.00 | 64% |
| Dysrhythmia | 1993-1999 | 62,991.00 | 5% |
|  | 2000-2009 | 403,859.00 | 31% |
|  | 2010-2019 | 830,468.00 | 64% |
| Congestive Heart Failure | 1993-1999 | 41,016.00 | 3% |
|  | 2000-2009 | 340,656.00 | 28% |
|  | 2010-2019 | 829,601.00 | 68% |
| Stroke | 1993-1999 | 19,861.00 | 7% |
|  | 2000-2009 | 102,205.00 | 34% |
|  | 2010-2019 | 177,560.00 | 59% |
| Peripheral Heart Disease | 1993-1999 | 8,726.00 | 3% |
|  | 2000-2009 | 75,278.00 | 30% |
|  | 2010-2019 | 170,274.00 | 67% |
| Myocardial Infarction | 1993-1999 | 13,850.00 | 8% |
|  | 2000-2009 | 64,066.00 | 37% |
|  | 2010-2019 | 96,359.00 | 55% |

Table S2. Interquartile Range of the Exposure Variables.

| **Temperature Variable** | **Fall** | | **Winter** | | **Spring** | | **Summer** | |
| --- | --- | --- | --- | --- | --- | --- | --- | --- |
|  | **Mean** | **IQR** | **Mean** | **IQR** | **Mean** | **IQR** | **Mean** | **IQR** |
| Minimum Temperature (°F) | 53.7 | 19.9 | 35.8 | 13.9 | 51.3 | 16 | 69.8 | 4 |
| Maximum Temperature (°F) | 75.5 | 14.9 | 58.1 | 14.9 | 75.1 | 12.2 | 89.5 | 5.9 |
| Mean Temperature (°F) | 63.8 | 16.7 | 46.1 | 13.6 | 62.9 | 14.1 | 78.7 | 5.3 |
| Temperature Variability | 12.5 | 4 | 13.3 | 3.9 | 13.7 | 3.7 | 11 | 2.4 |


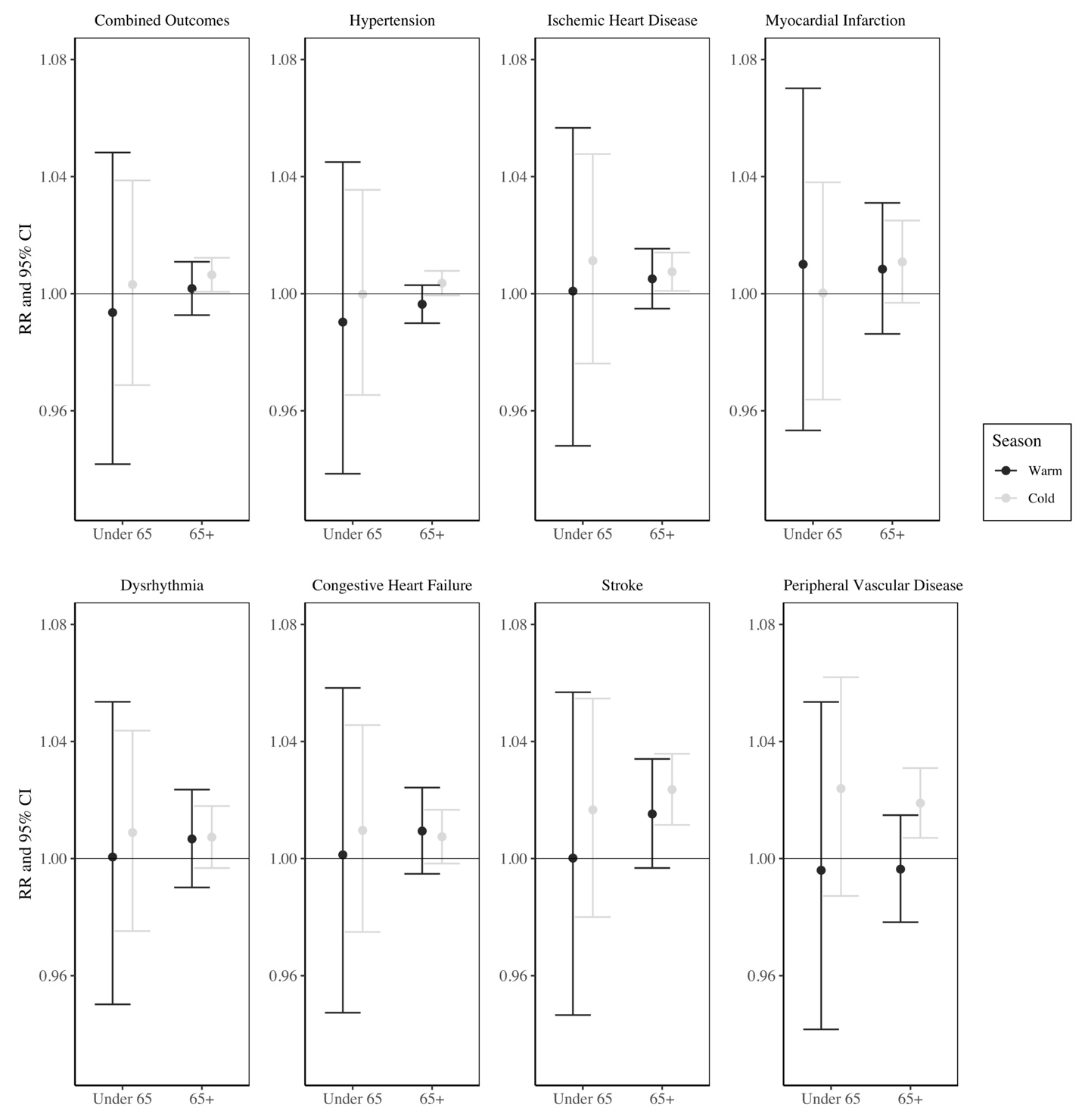


**Figure S1.** Relative risks and 95% confidence interval of an emergency department visits for each cardiovascular health outcome associated with an interquartile range increase in temperature variability from 1993-2019 in Atlanta, stratified by season (cold and warm) and age group (0-64 and 65+), and controlling for mean temperature, dew point temperature, time trend, day of the week, and holidays.

**
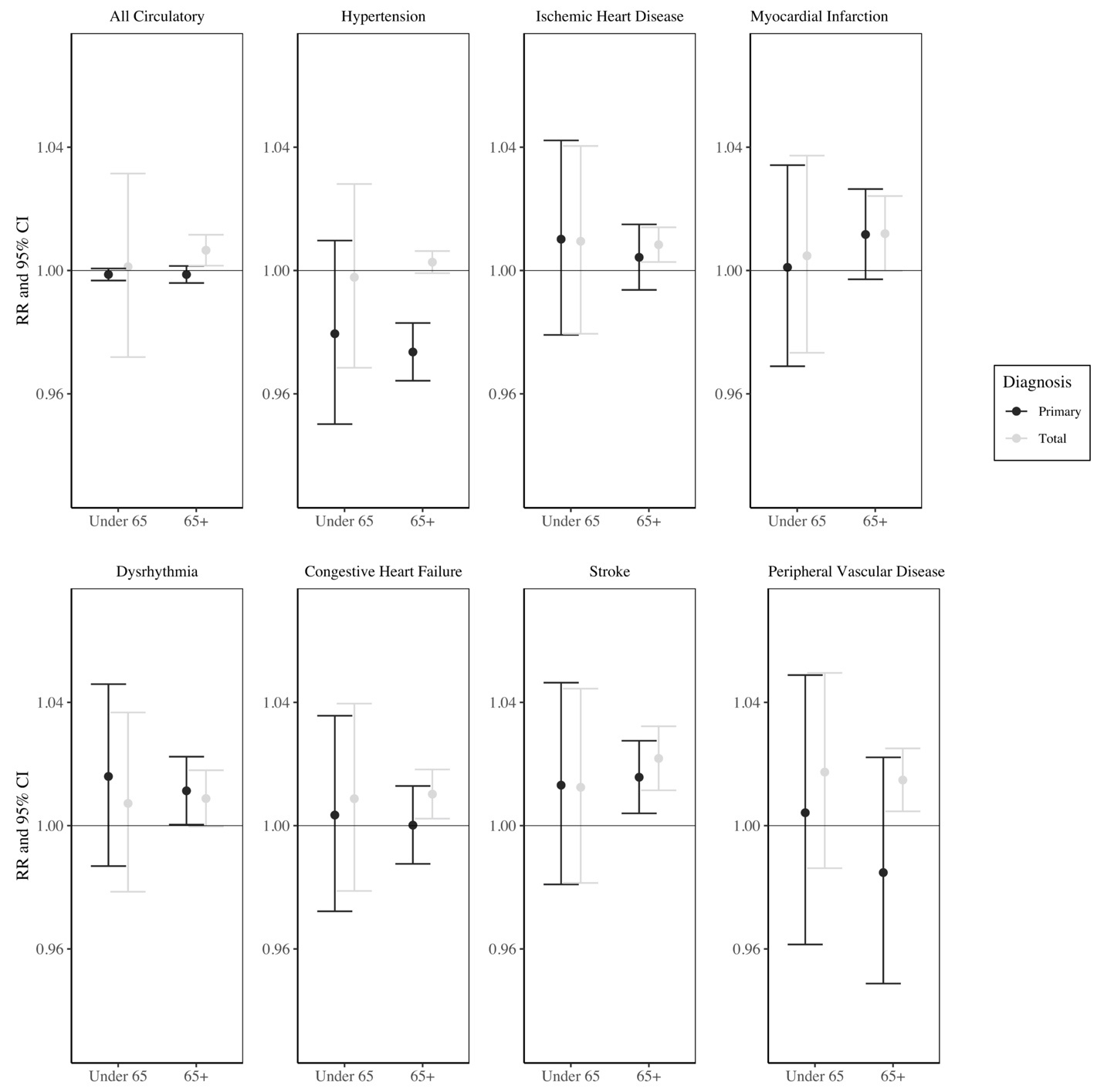
**

**Figure S2.** Relative risks and 95% confidence interval of an emergency department visits for each cardiovascular health outcome associated with an interquartile range increase in temperature variability from 1993-2019 in Atlanta, stratified by diagnosis type (primary only versus all diagnoses) and age group (0-64 and 65+), and controlling for mean temperature, dew point temperature, time trend, day of the week, and holidays.

**
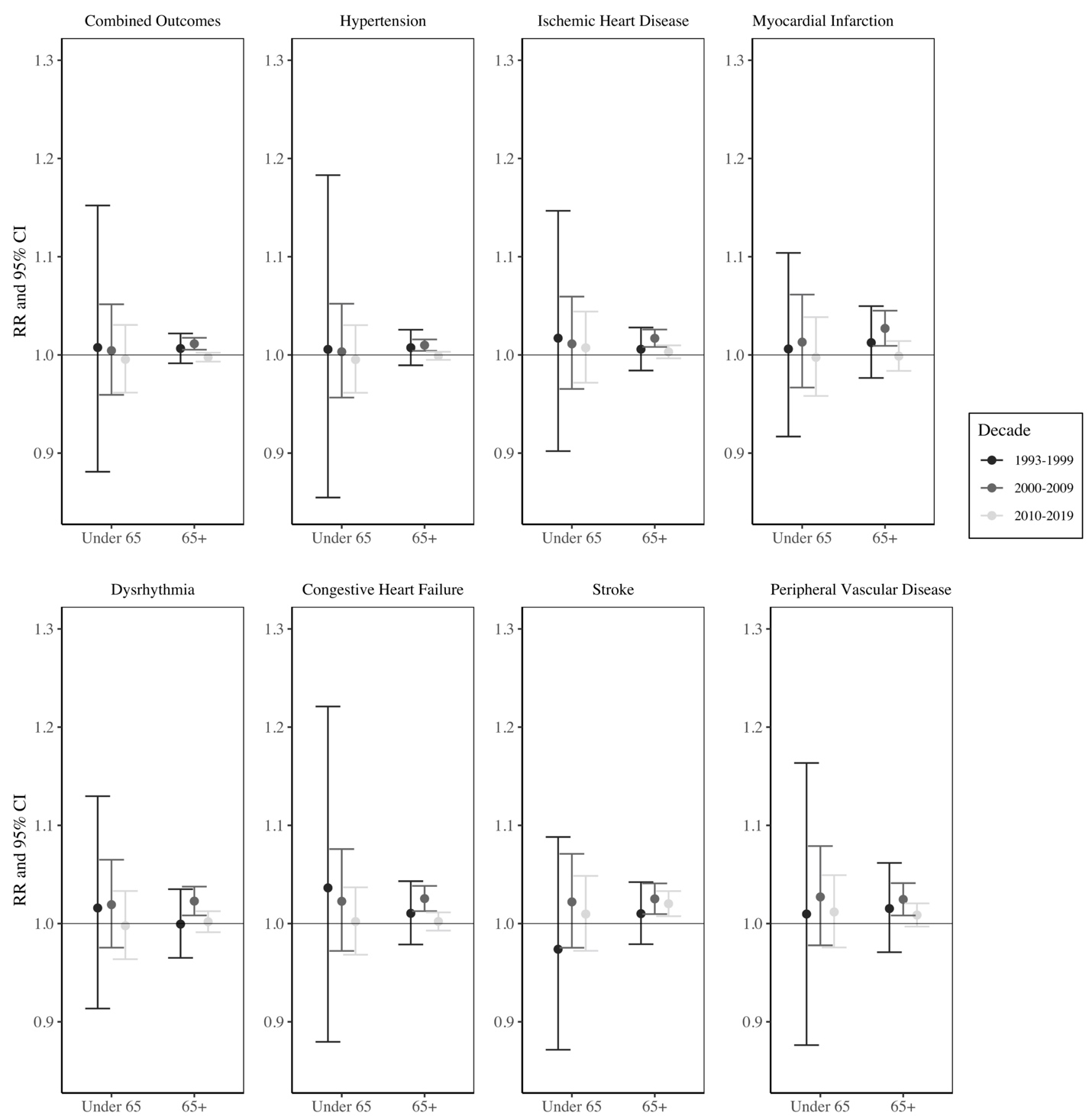
**

**Figure S3.** Relative risks and 95% confidence interval of an emergency department visits for each cardiovascular health outcome associated with an interquartile range increase in temperature variability from 1993-2019 in Atlanta, stratified by decade (1993-1999, 2000-2009, 2010-2019) and age group (0-64 and 65+), and controlling for mean temperature, dew point temperature, time trend, day of the week, and holidays.

**Sensitivity Analyses**

**
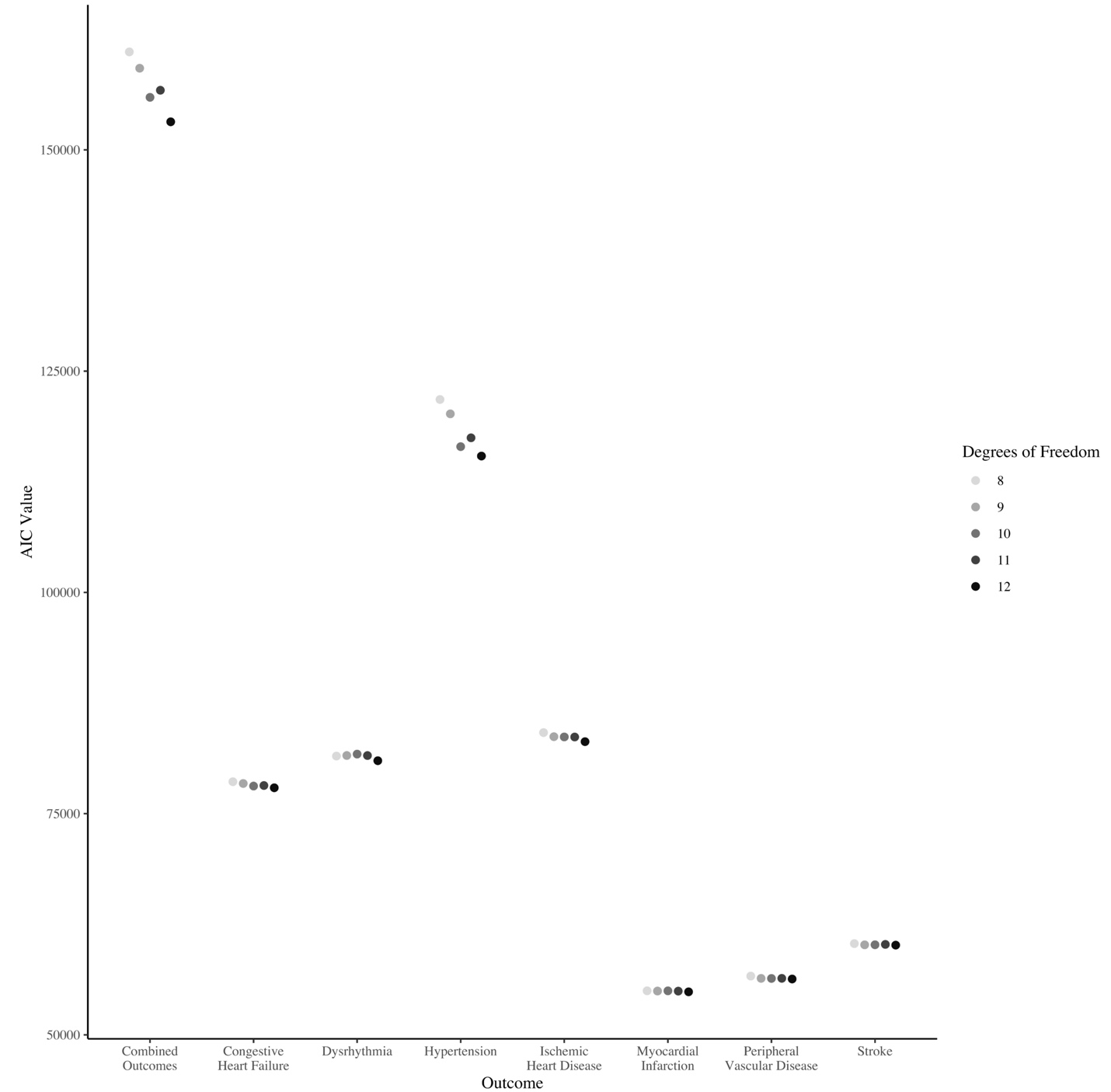
**

**Figure S4.** AIC values for the sensitivity analysis comparing varying degrees of freedom for the time spline.


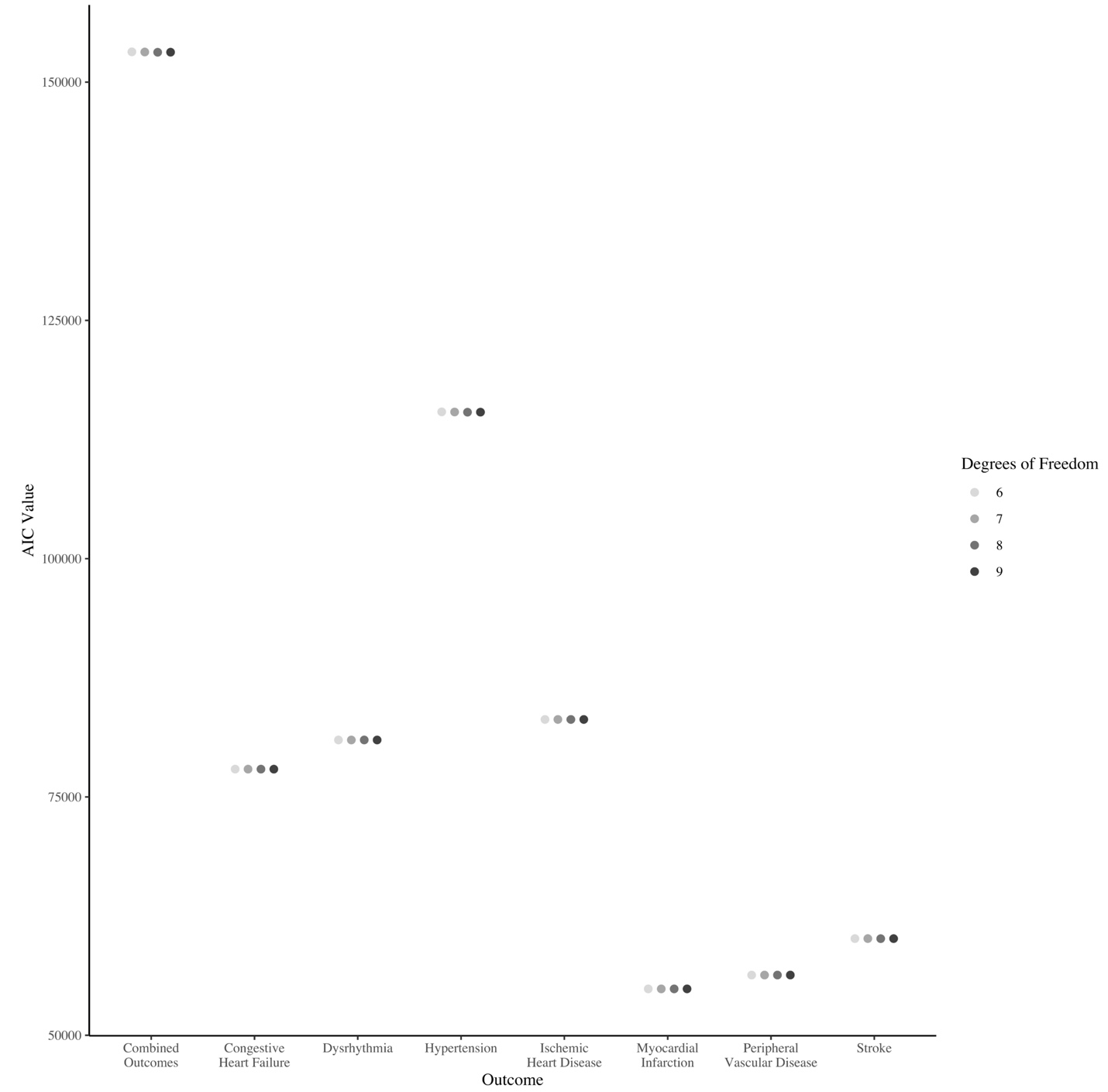


**Figure S5.** AIC values for the sensitivity analysis comparing varying degrees of freedom for the exposure spline.

**
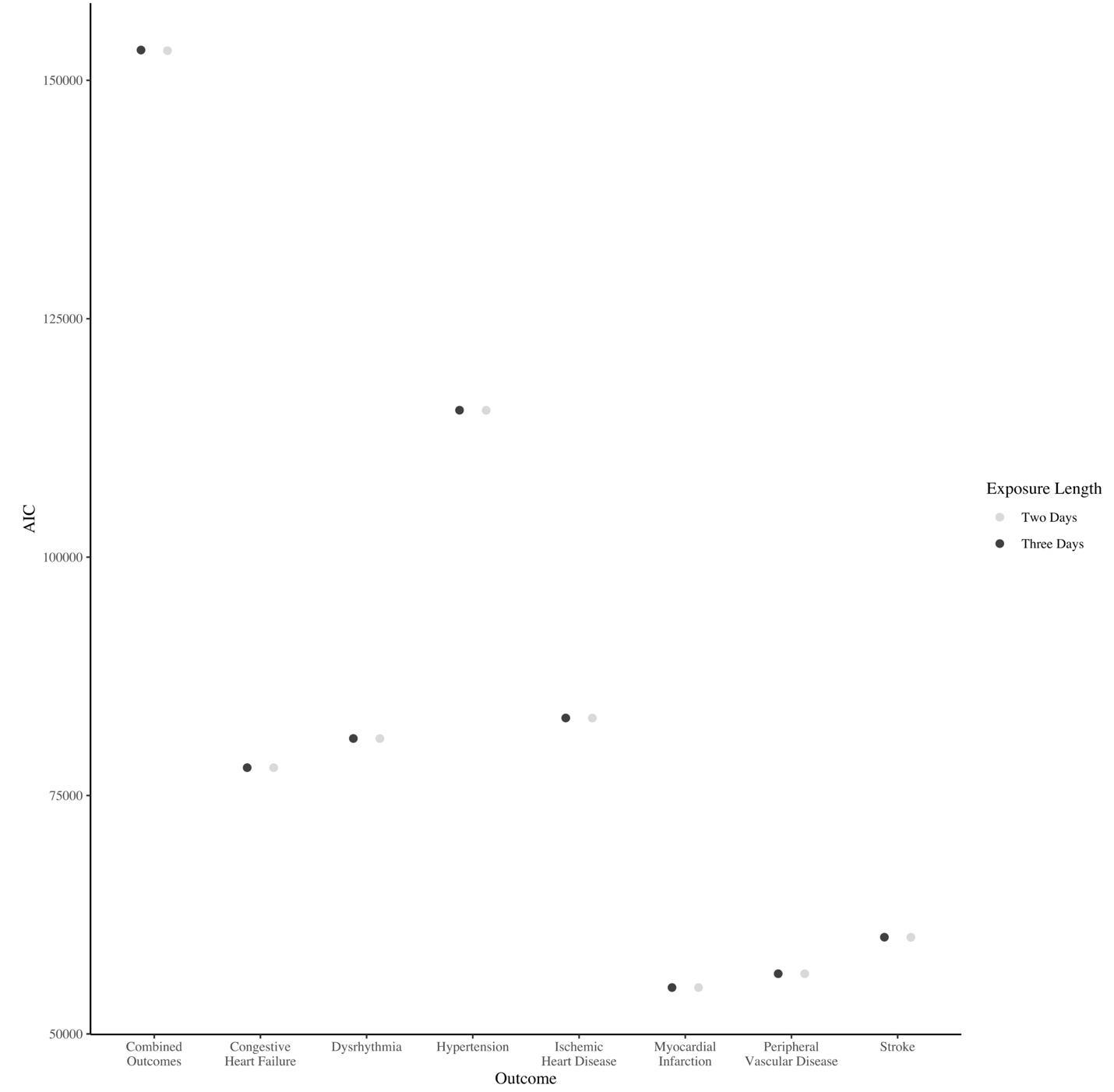
**

**Figure S6.** AIC values for the sensitivity analysis comparing varying day lengths of the temperature variability exposure.


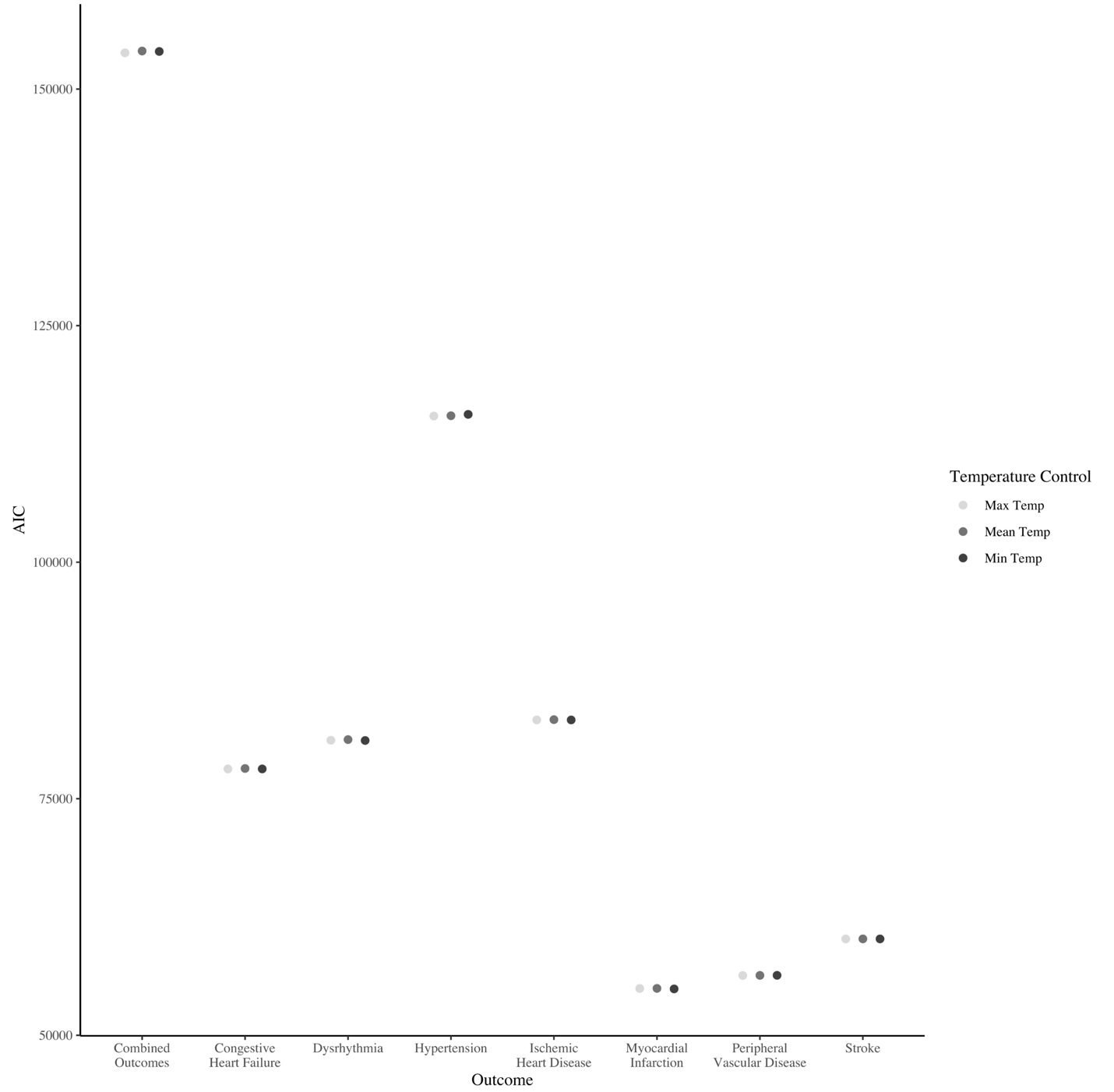


**Figure S7.** AIC values for the sensitivity analysis comparing varying three-day moving averages of temperature controls (maximum temperature, mean temperature, and minimum temperature).
